# The Relationship between MZ Generation Screen Golf Participants’ Participation Motivation, Self-Esteem, and Psychological Happiness

**DOI:** 10.3390/healthcare11182548

**Published:** 2023-09-14

**Authors:** Hyeon Jae Lee, Ji-Hye Yang, Chul-Ho Bum

**Affiliations:** 1 Department of Physical Education, Graduate School of Education, Kyung Hee University, Seocheon-dong 1, Giheung-gu, Yongin-si 17104, Gyeonggi-do, Republic of Korea; saintluclee@khu.ac.kr; 2 Department of Physical Education, Graduate School, Kyung Hee University, Seocheon-dong 1, Giheung-gu, Yongin-si 17104, Gyeonggi-do, Republic of Korea; didwlgp9@khu.ac.kr; 3 Department of Golf Industry, College of Physical Education, Kyung Hee University, Seocheon-dong 1, Giheung-gu, Yongin-si 17104, Gyeonggi-do, Republic of Korea

**Keywords:** MZ generation, participation motivation, self-esteem, psychological happiness

## Abstract

The purpose of this study is to analyze and clarify the relationship between the MZ generation’s participation motivation in screen golf, self-esteem, and psychological happiness. To reach the goals of this study, 300 MZ generation screen golf participants were selected for this study. Accordingly, a questionnaire was distributed and 275 questionnaires were used for this study, excluding the answers that were omitted or unfaithful. SPSS Version 29.0 was used to show the frequency analysis, exploratory factor analysis, correlation analysis, and multiple regression analysis of the research. The results of this study are as follows. First, it was found that the participation motivation of MZ generation screen golf participants had significant effects on positive self-esteem. Second, it was found that the participation motivation of MZ generation screen golf participants had significant effects on negative self-esteem. Third, it was found that the participation motivation of MZ generation screen golf participants had significant effects on psychological happiness. Fourth, it was found that the self-esteem of MZ generation screen golf participants had significant effects on psychological happiness. This study shows how to screen golf as part of a healthy leisure culture for the MZ generation and can enhance its psychological factors.

## 1. Introduction

### 1.1. Research Purpose and Significance

Society is continually changing, and interest in and the importance of leisure environments are rising day by day among these changes. For example, Evans [1] reported that a five-day workweek made most people feel less burdened about work time than in the past. As a result, it significantly affected people’s leisure time [2], and people naturally were considering how to spend their leisure time. According to reference [3], Korean people’s leisure time increased not only on weekdays but also on holidays during the five years from 2016 to 2021. 

With this increased interest in leisure, more people are using their leisure time for sports participation [4]. Golf is a sport that can enhance health among many leisure sports. It has the added benefit of participation in nature [5]. In addition, golf is less of a physical burden than other leisure sports, so it can be enjoyed regardless of age or sex. As such, golf is a sport that is worthy of study with respect to its effect on participants [6].

Golf rapidly became popular in Korea in the 1990s, and the number of golf courses increased [7], and the number of people who play golf significantly increased. However, several obstacles prevented its popularization [8]. Green fees rose above the inflation rate over the years, holding back the popularization of golf due to travel time and accessibility issues with golf courses [9]. The result was that golf, as a luxury sport, remained inaccessible to many people. 

Given this situation, and with advances in state-of-the-art technologies such as information technology (IT), golf simulators based on virtual reality (VR) emerged [10]. Using advanced technologies, golf simulators can present a virtual screen similar to the actual golf environment [11]. Additionally, since golf simulators are much cheaper and more affordable than an actual golf course, their popularity grew quickly [12] and garnered a lot of attention [13].

The emergence and growth of golf simulators has also led to more participation from the younger generations in their 20s and 30s, known as millennials and Generation Z (Generation MZ). Generation MZ is a collective term for millennials, born between 1980 and 1995, and Generation Z born afterward [14]. In Korea, Generation MZ’s percentage is over 30 percent of the total population [15], so they are considered important members. Interestingly, Generation MZ’s characteristics show differences compared to the other generations, such as baby boomers and generation X, who were born earlier than Generation MZ. In the case of Generation MZ, they appear more individualistic than the previous generation [16], and they have firm individual values [17]. Moreover, Iivari et al. [18] suggested that modern society has a generational digital gap because of technological advancement.

A number of previous studies that are related to Generation MZ have already been published in the field of sports [19], demonstrating that they quickly adapt to contemporary changes and are greatly influenced by new technologies such as VR [20], with one study [21] showing that VR sports games, such as golf simulators, can improve Generation MZ’s health and vitality. It seems that VR sports can significantly affect Generation MZ in modern society.

Furthermore, as social distancing was implemented, with leisure activities severely restricted during the COVID-19 pandemic in 2020 [22], most people in the world encountered social isolation because of the large-scale quarantine [23]. Moreover, the authors of Reference [24] reported that young adults, included in Generation MZ, also felt depression and symptoms of anxiety during these periods of isolation. Thus, the authors of Reference [25] reported that they need a method of releasing stress to solve this problem.

As a result, golf simulators undeniably became an attractive leisure activity for Generation MZ, a generation that is familiar and comfortable with the latest gadgets and technologies. Moreover, golf simulators’ strong advantages over real golf made Generation MZ more enthusiastic and motivated to participate in golf simulators than in real golf [26]. In addition to advantages like affordability and convenience, golf simulators have been found to have a positive psychological effect on Generation MZ participants. 

In psychology, self-esteem refers to the extent to which an individual believes he or she is worthy [27]. Collins et al. [28] found that sports may help participants improve their self-esteem, and Orth and Robins [29] reported that high self-esteem is an individual’s predictor of success and happiness. Furthermore, Dewar and Kavussanu [30] found that participation in golf positively affects participants’ happiness, demonstrating that Generation MZ participants can satisfy the desire for a happy life, a top priority for any individual, through golf simulators.

These positive effects have been recognized in the academic community, and studies have been conducted on golf simulators’ association with positive psychological factors [31,32] and their positive effects on Generation MZ participants [33]. Nevertheless, only a few studies focused on the relationship between Generation MZ’s participation motivation in golf simulators and their psychological happiness and self-esteem. Further, Generation MZ, by playing golf explosively in recent years, differ from previous generations. Hence, a study of golf-playing by generation in particular is also expected to show differences in terms of the relationship between participation motivation and psychological factors. 

Therefore, this study characterizes the effect of Generation MZ’s participation motivation in golf simulators on their self-esteem and psychological happiness. Moreover, it explores ways to improve the psychological life of Generation MZ through leisure activities.

### 1.2. Participation Motivation 

In sports, participation motivation represents an individual’s interest in or enjoyment of a sport [34], and it can be motivated by internal or external factors [35]. Furthermore, participation motivation is an important factor because it positively affects the understanding and analysis of how individuals want to participate in sports and the outcomes of their participation [36]. Thus, recent previous studies that are related to participation motivation have been continually published [37,38].

### 1.3. Self-Esteem

Self-esteem refers to the extent to which an individual feels he or she is worthy and evaluates himself or herself positively or negatively [39]. The authors of Reference [40] suggested that self-esteem is an emotional concept, which refers to a feeling about one’s existence, while self-concept is a cognitive concept formed about one’s existence. Furthermore, the authors of Reference [41] reported that self-esteem affects people’s overall life satisfaction. 

### 1.4. Psychological Happiness

Psychological happiness refers to a positive cognitive and affective emotional state arising from a combination of a variety of factors [42]. Medvedev and Landhuis [43] mentioned that psychological happiness is not a temporary emotion, such as joy, in peoples’ lives, but a continuous state. Moreover, people who play sports commonly enjoy sports to achieve psychological happiness [44].

Based on this intent, the hypotheses of this study are as follows:

1. Generation MZ’s participation motivation in golf simulators has a significant effect on their positive self-esteem.
2. Generation MZ’s participation motivation in golf simulators has a significant effect on their negative self-esteem.
3. Generation MZ’s participation motivation in golf simulators has a significant effect on their psychological happiness.
4. Generation MZ golf simulator participants’ self-esteem has a significant effect on their psychological happiness.

## 2. Materials and Methods

### 2.1. Participants and Procedure

This study conducted both online and offline questionnaire surveys by distributing questionnaire copies to 300 Generation MZ respondents who participated in golf simulators and lived in Seoul or Gyeonggi-do. After getting the participants’ oral voluntary agreement, we proceed with the questionnaires which were collected immediately after they were filled in. In the case of the online questionnaires, this sentence—‘Your participation in this study is completely voluntary. There are no foreseeable risks associated with this study. However, if you feel uncomfortable answering any questions, you can withdraw from the survey at any point’—was filled in the questionnaire, so all respondents voluntarily progressed with the questionnaires. The survey was conducted from 1 March to 14 April 2023. After collecting the questionnaire copies (online 160, offline 140) and excluding 25 copies with incomplete or missing responses, 275 copies were finally selected as valid and were analyzed. The sample was extracted using the convenient sampling method, which is one of the non-probability sampling methods, and the questionnaire was self-administered by the respondents. Table 1 shows the respondents’ demographic characteristics (gender, age, highest level of education achievement, employment, using time per day, frequency of playing golf).

### 2.2. Measures

This study used a questionnaire as a tool to investigate the relationship between Generation MZ golf simulator participants’ participation motivation, self-esteem, and psychological happiness. For the participation motivation questionnaire, the sport participation motivation scale by Kim [45] was used, which was modified and developed by Jung [46] based on the Sport Motivation Scale (SMS-28) by Vallerand et al. [47], which was used in a recent study by Rosario [48]. The Leisure Intrinsic Motivation (LIM) by Weissinger and Banders [49] was slightly adapted by excluding the condition factor in intrinsic motivation to accommodate the characteristics of this study. For the self-esteem questionnaire, the Self-Esteem Scale (SES) developed by Rosenberg [50] and used by Kim et al. [51] was adapted. For the psychological happiness questionnaire, the psychological happiness scale developed by Yang [52], based on the Personality Expressive Activities Questionnaire (PEAQ) by Waterman [53] and the sub-factors of the Scale of Psychological Well-being by Ryff [54] and used by Jeong [55] were adapted to accommodate the characteristics of this study. All factors were based on a five-point Likert scale (1 = Strongly disagree, 5 = Strongly agree). 

### 2.3. Statistical Data Analysis

This study used the IBM SPSS Statistics Version 29.0 used in previous studies that were related to the sports, such as References [56,57], and statistical analysis was conducted as follows. First, frequency analysis was conducted to analyze the demographic characteristics of the Generation MZ golf simulator participants. Second, exploratory factor analysis was conducted to verify the validity of the questionnaire, which was used as a tool in this study. Third, to verify the reliability of the questionnaire, this study used Cronbach’s alpha, of which the minimum value of the factorial loads is α = 0.7. Fourth, Pearson correlation analysis was conducted to determine the relationship between the variables. Fifth, multiple regression analysis was conducted to determine the relationship between Generation MZ’s participation motivation in golf simulators, self-esteem, and psychological happiness. The level of significance considered in the analysis is *p* < 0.05.

## 3. Results 

### 3.1. Scale Validity and Reliability

This study conducted an exploratory factor analysis on the questionnaire items for participation motivation, self-esteem, and psychological happiness to ensure the validity of the questionnaire. Principal component analysis was used as the method to extract factors, and the varimax method was used as the factor rotation method. 

The study found that participation motivation had seven sub-factors (no motivation, health and physical fitness, enjoyment, skill development, socialization, external appeal, achievement). No motivation and enjoyment had five items, while health and physical fitness, skill development, socialization, and external appeal had four items, and achievement had three items. Self-esteem had two sub-factors (positive self-esteem and negative self-esteem), both with five items. Psychological happiness had one factor with five items. Table 2, Table 3 and Table 4 provide more details.

The reliability of the questionnaire was tested using Cronbach’s alpha, which was also used to test for internal consistency reliability. According to Barbera et al. [58], a common satisfactory reliability load is α = 0.7, so the results were verified based on this value. As a result, all the results showed satisfactory reliability (No motivation: α = 0.946, Health and physical fitness: α = 0.948, Enjoyment: α = 0.909, Skill development: α = 0.920, Socialization: α = 0.899, External appeal: α = 0.885, Achievement: α = 0.902, Positive self-esteem: α = 0.913, Negative self-esteem: α = 0.880, Psychological happiness: α = 0.882). Thus, the result shows that the questionnaire’s validity and reliability are appropriate to use.

### 3.2. Correlation Analysis

The correlation results revealed that the correlation between all variables, except for those between health and physical fitness and positive self-esteem and between health and physical fitness and negative self-esteem, were statistically significant. Moreover, the strongest correlation is between positive self-esteem and psychological happiness (*r* = 0.782, *p* < 0.01). Table 5 shows more details. 

### 3.3. Multiple Regression Analysis 

Regarding the effect of participation motivation on positive self-esteem among Generation MZ golf simulator participants, enjoyment (β = 0.189, *t* = 2.349, *p* < 0.05), achievement (β = 0.123, *t* = 2.192, *p* < 0.05), and socialization (β = 0.207, *t* = 3.509, *p* < 0.01), among the other among sub-factors of participation motivation, positively affected positive self-esteem. Regarding the results about the effect of participation motivation on negative self-esteem, skill development (β = −0.197, *t* = −2.163, *p* < 0.05), a sub-factor of participation motivation, negatively affected negative self-esteem. Additionally, regarding the effect of participation motivation on psychological happiness, skill development (β = 0.279, *t* = 3.566, *p* < 0.001), enjoyment (β = 0.182, *t* = 2.274, *p* < 0.05), and socialization (β = 0.180, *t* = 3.181, *p* < 0.01), sub-factors of participation motivation, positively affected psychological happiness. Finally, regarding the effect of self-esteem on psychological happiness, positive self-esteem (β = 0.704, *t* = 17.936, *p* < 0.001), a sub-factor of self-esteem, positively affected psychological happiness, and negative self-esteem (β = −0.200, *t* = −5.094, *p* < 0.001), the other sub-factor of self-esteem, negatively affected psychological happiness. Table 6, Table 7, Table 8 and Table 9 show more details about the above results. 

## 4. Discussion

The results of this study show that enjoyment, achievement, and socialization among participation motivation positively affected positive self-esteem. However, skill development, one of the participation motivation sub-factors, negatively affected negative self-esteem. Moreover, skill development, enjoyment, and socialization among participation motivation positively affected psychological happiness. Finally, positive self-esteem positively affected psychological happiness, but negative self-esteem negatively affected psychological happiness.

Previous studies verified the statistical significance for the positive effect of enjoyment, achievement, and socialization, sub-factors of participation motivation, on positive self-esteem, and the negative effect of skill development, a sub-factor of participation motivation, on negative self-esteem. For example, Sattar et al. [59] reported that participants spending more time in sports with a high level of participation motivation had a higher level of self-esteem. Qurban et al. [60] noted that these two factors were closely psychologically related, which is consistent with the results of this study. Furthermore, the negative effect of skill development on negative self-esteem means that more participation based on skill development leads to a higher level of self-esteem, which is consistent with the results reported by the authors of Reference [61] that skill development and achievement improve positive self-esteem and subsequently resolve negative self-esteem.

Further, skill development, enjoyment, and socialization, sub-factors of participation motivation, had a positive effect on psychological happiness, which is consistent with the results of Reference [62] that participation motivation in sports where participants feel enjoyment, socialization, and external appeal affects their happiness. Moreover, it is partially consistent with the results of Reference [63], that internal motivation and no motivation affects psychological happiness, and those of Reference [64], that external motivation affects psychological happiness. As this study combined the internal motivation sub-factors of skill development, enjoyment, and achievement, and the external motivation sub-factors of socialization, health, and physical fitness as well as external appeal into the sub-factors of participation motivation, this categorization may have caused some partial differences in the results.

Finally, positive self-esteem, a sub-factor of self-esteem, positively affected psychological happiness, and negative self-esteem, the other sub-factor of self-esteem, negatively affected psychological happiness. Many studies reported the statistical significance of self-esteem and psychological happiness. Oh [65] found that adult female sports participants’ positive self-esteem positively affects their psychological happiness and their negative self-esteem negatively affects their psychological happiness, which is consistent with this study’s results. 

Consequently, golf simulators positively affected psychological happiness by helping Generation MZ to improve self-esteem and have a high level of perception, as suggested by previous findings indicating that improved self-esteem through leisure sport activities may increase psychological happiness. Thus, this finding is important as it shows that Generation MZ and golf simulators have a significant relationship, and that these simulators can be used to improve their psychological life.

## 5. Conclusions

Based on the results of this study, Generation MZ continually prefers playing golf simulators because they have digital characteristics with which they are familiar and they adapt well to the changing environment. Moreover, Generation MZ encountered COVID-19 limitations, so they discovered golf simulators’ additional attraction. As a result, this study found Generation MZ enhances their psychological health through enjoying golf simulators. 

It can also be concluded that participation in golf simulators, for the purpose of improving internal values based on self-determination, such as skill development and enjoyment, or bonding with others, positively affects an individual’s self-esteem and psychological happiness. As Generation MZ’s interest in and importance afforded to leisure sports grow day by day, this study would serve as a good reference for providing directions on what kind of mindset Generation MZ need to have when using golf simulators. Participation in golf simulators with the right mindset would be a good way to bring stability to the lives of individuals who do not know what to pursue in the early days of their career, and overcome COVID-19 problems such as depression and isolation.

## 6. Limitations

This study was conducted to examine the relationship between Generation MZ’s participation motivation in golf simulators, self-esteem, and psychological happiness. Based on the process and results of the study, the authors would like to suggest the following:

First, since this study focused only on Generation MZ golf simulator participants, it is difficult to generalize its results to all leisure sports. Thus, it would be necessary to conduct an expanded follow-up study on various other sports.

Second, since this study limited its population to Generation MZ golf simulator participants in Seoul, Gyeonggi-do, and the Seoul metropolitan area, it is difficult to generalize its results to Generation MZ golf simulator participants across all other regions. Hence, it would be important to conduct a follow-up study on more regions.

Third, this study focused on quantitative research on the relationship between Generation MZ’s participation motivation in golf simulators, self-esteem, and psychological happiness. Therefore, follow-up studies using in-depth interviews would produce more useful results.

Fourth, this study only focused on Generation MZ participants, making it difficult to generalize the results to all age groups of golf simulator users. Therefore, it would be important to conduct a follow-up study on those older or younger than Generation MZ.

## Figures and Tables

**Table 1:** Study participants’ social demographic information.

Demographic		Frequency	Percentage (%)
Gender	Male	177	64.4
	Female	98	35.6
Age	20–29	153	55.6
	30–39	79	28.7
	40–45	43	15.6
Highest level of educational achievement	High school and below	8	2.9
	Attending university	98	35.6
	Bachelor’s	143	52.0
	Master’s and above	26	9.5
Employment	Office job	54	19.6
	Technical	21	7.6
	Professional	40	14.5
	Self-employed	28	10.2
	Student	110	40.0
	Other	22	8.0
Using time per day	Less than 1 h	111	40.4
	Less than 1–2 h	104	37.8
	Greater than 2 h	60	21.8
Frequency of playing golf	1–2 times per week	211	76.7
	3–4 times per week	53	19.3
	5–6 times per week	11	4.0
	Total	275	100

**Table 2:** Results of Exploratory Factor Analysis for participation motivation.

Items	1	2	3	4	5	6	7
No motivation 28	0.887	0.083	−0.085	−0.117	−0.079	−0.157	−0.078
No motivation 29	0.883	0.114	−0.140	−0.059	−0.055	−0.098	−0.069
No motivation 26	0.867	0.134	−0.053	−0.138	−0.131	−0.044	−0.057
No motivation 27	0.864	0.098	−0.203	−0.119	−0.107	−0.127	−0.113
No motivation 25	0.861	0.092	−0.122	−0.119	−0.131	−0.006	−0.033
Health and physical fitness 18	0.115	0.915	0.103	0.159	−0.004	0.102	−0.006
Health and physical fitness 19	0.159	0.910	0.178	0.131	0.025	0.126	0.035
Health and physical fitness 17	0.153	0.906	0.111	0.116	0.022	0.145	0.041
Health and physical fitness 20	0.085	0.852	0.041	0.092	0.143	0.142	0.149
Enjoyment 6	−0.169	0.093	0.784	0.317	0.188	0.096	0.170
Enjoyment 7	−0.154	0.119	0.782	0.252	0.231	0.167	0.192
Enjoyment 8	−0.115	0.113	0.727	0.330	0.176	0.207	0.203
Enjoyment 5	−0.242	0.008	0.697	0.082	0.318	0.020	0.265
Enjoyment 9	−0.146	0.211	0.586	433	0.101	0.161	0.220
Skill development 2	−0.126	0.165	0.268	0.835	0.099	0.115	0.182
Skill development 1	−0.260	0.167	0.226	0.805	0.147	0.040	0.165
Skill development 3	−0.136	0.099	0.266	0.772	0.144	0.172	0.269
Skill development 4	−0.125	0.240	0.305	0.656	0.085	0.167	0.341
Socialization 14	−0.086	0.045	0.201	0.069	0.865	0.180	0.067
Socialization 15	−0.114	0.071	0.145	0.093	0.853	0.124	0.082
Socialization 13	−0.051	0.087	0.146	0.121	0.832	0.170	0.038
Socialization 16	−0.222	−0.022	0.167	0.104	0.763	0.057	0.184
External appeal 23	−0.142	0.115	0.055	0.078	0.158	0.855	0.108
External appeal 24	−0.025	0.257	0.051	0.150	0.066	0.829	−0.026
External appeal 22	−0.096	138	0.216	0.089	0.106	0.794	0.165
External appeal 21	−0.132	0.030	0.116	0.077	0.214	0.784	0.176
Achievement 10	−0.144	0.066	0.288	0.235	0.156	0.143	0.805
Achievement 12	−0.117	0.134	0.254	0.254	0.120	0.139	0.787
Achievement 11	−0.070	0.021	0.254	0.368	0.132	0.184	0.765
Eigenvalues	4.298	3.609	3.375	3.305	3.261	3.114	2.498
Variance (%)	14.821	12.444	11.639	11.396	11.245	10.738	8.615

**Table 3:** Results of Exploratory Factor Analysis for self-esteem.

Items	1	2
Positive self-esteem 4	0.869	−0.162
Positive self-esteem 3	0.856	−0.203
Positive self-esteem 2	0.855	−0.202
Positive self-esteem 1	0.845	−0.173
Positive self-esteem 5	0.792	−0.125
Negative self-esteem 6	−0.124	0.843
Negative self-esteem 8	−0.187	0.840
Negative self-esteem 10	−0.157	0.817
Negative self-esteem 7	−0.253	0.795
Negative self-esteem 9	−0.125	0.762
Eigenvalues	3.717	3.451
Variance (%)	37.169	34.512

**Table 4:** Results of Exploratory Factor Analysis for psychological happiness.

Items	1
Psychological happiness 3	0.865
Psychological happiness 1	0.829
Psychological happiness 4	0.829
Psychological happiness 2	0.809
Psychological happiness 5	0.793
Eigenvalues	3.405
Variance (%)	68.099

**Table 5:** Results of Correlation analysis.

Items	1	2	3	4	5	6	7	8	9	10
1	1									
2	0.706 **	1								
3	0.656 **	0.651 **	1							
4	0.355 **	0.495 **	0.368 **	1						
5	0.339 **	0.265 **	0.207 **	0.134 *	1					
6	0.371 **	0.396 **	0.382 **	0.358 **	0.313 **	1				
7	−0.341 **	−0.379 **	−0.284 **	−0.289 **	0.194 **	−0.233 **	1			
8	0.448 **	0.502 **	0.456 **	0.424 **	0.053	0.274 **	−0.317 **	1		
9	−0.284 **	−0.245 **	−0.229 **	−0.140 *	−0.053	−0.142 *	0.174 **	−0.393 **	1	
10	0.516 **	0.515 **	0.425 **	0.409 **	0.199 **	0.326 **	−0.231 **	0.782 **	−0.477 **	1

Note. ** *p* < 0.01, * *p* < 0.05, 1 = Skill development, 2 = Enjoyment, 3 = Achievement, 4 = Socialization, 5 = Health and physical fitness, 6 = External appeal, 7 = No motivation, 8 = Positive self-esteem, 9 = Negative self-esteem, 10 = Psychological happiness.

**Table 6:** The effect of participation motivation on positive self-esteem.

Dependent Variables		B	SE	β	*t*	*p*
Positive self-esteem	Skill development	0.103	0.058	0.138	1.755	0.080
	Enjoyment	0.162	0.069	0.189	2.349	0.020 *
	Achievement	0.123	0.056	0.156	2.192	0.029 *
	Socialization	0.185	0.053	0.207	3.509	0.001 **
	Health and physical fitness	−0.057	0.034	−0.100	−1.691	0.092
	External appeal	0.021	0.042	0.029	0.503	0.616
	No motivation	0.040	0.035	−0.067	−1.135	0.258
*F* = 19.633, *R*^2^ = 0.340, Adjusted *R*^2^ = 0.323						

Note. * *p* < 0.05, ** *p* < 0.01.

**Table 7:** The effect of participation motivation on negative self-esteem.

Dependent Variables		B	SE	β	*t*	*p*
Negative self-esteem	Skill development	−0.197	0.091	−0.200	−2.163	0.031 *
	Enjoyment	−0.054	0.107	−0.048	−0.506	0.613
	Achievement	−0.047	0.087	−0.045	−0.539	0.590
	Socialization	−0.008	0.082	−0.007	−0.102	0.919
	Health and physical fitness	0.026	0.052	0.034	0.496	0.620
	External appeal	−0.024	0.065	−0.025	−0.371	0.711
	No motivation	0.047	0.055	0.060	0.862	0.389
*F* = 3.865, *R*^2^ = 0.092, Adjusted *R*^2^ = 0.068						

Note. * *p* < 0.05.

**Table 8:** The effect of participation motivation on psychological happiness.

Dependent Variables		B	SE	β	*t*	*p*
Psychological happiness	Skill development	0.224	0.063	0.279	3.556	0.000 ***
	Enjoyment	0.169	0.074	0.182	2.274	0.024 *
	Achievement	0.025	0.060	0.030	0.417	0.677
	Socialization	0.180	0.057	0.186	3.181	0.002 **
	Health and physical fitness	0.000	0.036	0.000	−0.008	0.994
	External appeal	0.058	0.045	0.075	1.291	0.198
	No motivation	0.008	0.038	0.013	0.213	0.831
*F* = 20.381, *R*^2^ = 0.348, Adjusted *R*^2^ = 0.331						

Note. * *p* < 0.05, ** *p* < 0.01, *** *p* < 0.001.

**Table 9:** The effect of self-esteem on psychological happiness.

Dependent Variables		B	SE	β	*t*	*p*
Psychological happiness	Positive self-esteem	0.761	0.042	0.704	17.936	0.000 ***
	Negative self-esteem	−0.163	0.032	−0.200	−5.094	0.000 ***
*F* = 248.070, *R*^2^ = 0.646, Adjusted *R*^2^ = 0.643						

Note. *** *p* < 0.001.

## Data Availability

Not appliable.
